# Socioeconomic trajectory from birth to adolescence and lung function: prospective birth cohort study

**DOI:** 10.1186/1471-2458-11-596

**Published:** 2011-07-27

**Authors:** Ana MB Menezes, Samuel C Dumith, Rogélio Perez-Padilla, Ricardo B Noal, Fernando C Wehrmeister, Jeovany Martínez-Mesa, Cora LP Araújo, Pedro C Hallal

**Affiliations:** 1 Postgraduate Program in Epidemiology, Federal University of Pelotas, Pelotas, Brazil; 2 Postgraduate Program in Epidemiology, and Physical Activity Epidemiology Research Group, Federal University of Pelotas, Pelotas, Brazil; 3 National Institute of Respiratory Diseases, Mexico City, Mexico

**Keywords:** Spirometry, socioeconomic factors, adolescent, cohort studies, developing countries

## Abstract

**Background:**

Socioeconomic status (SES) has been shown to be an important contributor to lung function. The aim of this study was to evaluate the association between lung function in adolescence and (a) SES at birth; (b) SES in adolescence; (c) SES trajectory from birth to adolescence ('never poor', 'non poor-poor', 'poor-non poor' and 'always poor'). Additionally, we investigate the role of adolescent and parental variables at mediating these associations.

**Methods:**

Prospective birth cohort study in Pelotas, Brazil, including 4,005 adolescents (mean age: 14.7 years) followed up from birth. Lung function was measured by spirometry. Outcome variables were forced expiratory volume in one second in liters (FEV1) and forced vital capacity also in liters (FVC).

**Results:**

Mean FEV1 was 3.46 L (95%CI 3.43-3.49) among boys and 2.93 L (95%CI 2.91-2.95) among girls. Mean FVC was 4.00 L (95%CI 3.97; 4.04) among boys and 3.30 L (95%CI 3.27; 3.32) among girls. SES at birth, in adolescence and its trajectory from birth to adolescence were inversely associated with lung function in both adolescent boys and girls. After adjustment for mediating variables, coefficients were largely reduced, particularly among boys, and the main predictor of change in coefficients was the inclusion of height in the models.

**Conclusion:**

Low income adolescents from Brazil present impaired lung function as compared to the better off, and this is largely explained by height.

## Background

Socioeconomic status (SES) has been shown to be an important contributor to lung function. In a systematic review of articles published over the past 20 years, Hegewald and Crapo showed that poverty in adults was related to a reduction of > 300 mL in forced expired volume in one second (FEV1) among men and > 200 mL among women [1]. As compared to studies among adults, those including children and adolescents were less frequent in the literature. In addition, most studies were cross-sectional and were carried out in high-income countries.

Most prospective studies in the field have evaluated the roles of birthweight and respiratory infections in infancy and childhood on later lung function [2]. Those analyzing SES and later lung function used static measures of SES at a given age [3]. We were unable to locate articles describing the long-term association between SES trajectories during the life course and lung function.

The aims of this article were to evaluate the association between lung function in adolescence, expressed by FEV1 and forced vital capacity (FVC), and (a) socioeconomic status at birth; (b) socioeconomic status in adolescence; (c) socioeconomic trajectory from birth to adolescence. Additionally, we investigate the role of sociodemographic, anthropometric, behavioral and parental variables at mediating these associations.

## Methods

All hospital-born children in 1993 (N = 5,265) residents in the city of Pelotas, Southern Brazil were eligible for a birth cohort study; there were only 16 refusals [4,5]. At the age of 14-15 years all cohort participants were visited at home and a questionnaire was administered. Adolescents were also invited to visit the "Research Clinic" for performing spirometry.

Pulmonary function was evaluated with the adolescents seated, using a noseclip and a disposable mouthpiece. A portable, battery-operated, ultrasound transit-time based spirometer (*Easy-One2; NDD Medical Technologies, Chelmsford MA, USA and Zurich, Swizerland*) was used. Exclusion criteria for spirometry included a positive answer for any of the following questions in the last three months: thoracic or abdominal surgery, heart problem, eye surgery and admission to hospital for any cardiac condition; pregnancy among the girls was also exclusion for spirometry. By using these exclusion criteria, 64 adolescents were ineligible to perform spirometry. Adolescents performed as many forced expiratory maneuvers as needed in order to produce three acceptable and repeatable. Over 90% of all spirometric tests fulfilled the 2005 American Thoracic Society-European Respiratory Society quality criteria [6]. Quality of all tests was assessed centrally by one person (RPP).

Outcome variables were forced expiratory volume in one second in liters (FEV1) and forced vital capacity also in liters (FVC). Socioeconomic level was based on family income collected at birth and at 14-15 years of age, and was categorized in tertiles for the analyses. Mean family income at the 15 years follow up visit was around U$ 200 for the lowest tertile, U$ 500 for the intermediate tertile and U$ 1500 for the wealthiest tertile. Mean income in Pelotas is slightly higher than the national average. However, because Brazil is a big country with important income inequalities, the mean family income in Pelotas is much lower than that of the wealthiest cities in the South and Southeast regions, but much higher than that of most cities of the Northeast and North regions.

Socioeconomic trajectories were created based on the combination of socioeconomic levels at birth and at 14-15 years of age. Adolescents who were classified in the lowest tertile in both periods were categorized as 'always poor'. Those who were classified in the lowest tertile at birth, but in the intermediate or top tertile at 14-15 years of age were categorized as 'poor, non-poor". Similarly, those classified in the intermediate or top tertile at birth and at the bottom tertile at 14-15 years were categorized as "non-poor, poor". Finally, those classified in both visits in the top tertile were categorized as "never poor". Family income at birth and at 14-15 years of age was measured in the same way at these two data collection points and it was based on parental report.

The mediating variables included in this paper were: height at 14-15 years (measured to the nearest 0.1 cm using a locally made portable stadiometer), weight at 14-15 years (measured to the nearest 100 g using an electronic SECA weight scale), adolescent skin color (self reported and categorized as white, mixed or black), pubertal stage by Tanner stages [7], medical diagnosis of allergy (yes/no), parental smoking when adolescents were aged 11 years (yes if any of the parents reported it vs. none), parental wheezing when adolescent were 11 years of age (presence of wheezing within the last 12 months either for the mother or father vs. none), adolescent self-reported smoking at 14-15 years of age, adolescent wheezing at 14-15 years of age (wheezing within the last 12 months), age at follow-up and physical activity in minutes per week (calculated using a validated questionnaire on leisure-time and commuting physical activity) [8].

Prior to data collection, standardization sessions for the anthropometric measurements were carried out. These sessions were repeated every two months during the fieldwork. Reported measurement errors from the National Center for Health Statistics were used as the acceptable limits in these standardization sessions. Approximately 10% of the interviewees were re-visited by a field supervisor one or two weeks after the original interview, and a short version of the entire questionnaire was administered for quality control purposes.

Data were analyzed using Stata 10.0 (*StataCorp, College Station, Texas, USA*). Descriptive analyses included calculation of means and standard deviations for pulmonary function parameters stratified by sex. In the unadjusted analysis, simple linear regression was used to compare FEV1 and FVC according to socioeconomic variables. Multivariable analyses included linear regression using two different models. Model one included current age, height and weight separated for each gender. Model 2 added to model 1 adjustment by parental (smoking and wheezing) and adolescent variables (wheezing, smoking, pubertal stage, physical activity, skin color and allergy).

In order to analyze the mediating effect of several variables on the association between socioeconomic status and lung function, we ran models including one extra variable at a time, and examined the change in beta coefficients and coefficient of determination (R²). The order in which mediating variables were included in the model was defined by the significance of its association with lung function. Significance level was set at 5% for two-tailed tests.

Further details on the methodology of the 1993 Pelotas (Brazil) birth cohort study can be found elsewhere [4,5]. All phases of the 1993 Pelotas birth cohort study were approved by the Medical School Ethics Committee of the Federal University of Pelotas [process 158/07]. Written informed consent was obtained prior to each follow-up.

## Results

The original cohort included 5,249 children born in 1993 in Pelotas, Brazil. From birth to 14-15 years of age, 148 cohort members were known to have died. We interviewed 4,325 adolescents, thus totaling a response rate of 85.7%. Out of the 4,325 subjects located, 4,005 provided valid spirometric data. The mean age at follow-up was 14.7 years (SD = 0.3); 51% of them were female, 64% self-rated their skin color as white, 27% were overweight, 12% reported wheezing in the past year, 39% presented medical diagnosis of rhinitis or eczema, and 19% reported to have ever smoked. Exactly half of them had at least one parent who was current smoker, and 34% of parents referred to present asthma. Mean body weight and height were, respectively, 57.4 kg (SD = 12.7) and 163.2 cm (SD = 8.2).

The mean FEV1 for the whole sample was 3.19 l (95%CI 3.17-3.21), being 3.46 l (95%CI 3.43-3.49) among boys and 2.93 l (95%CI 2.91-2.95) among girls. Mean FVC was 3.65 l (95%CI 3.62-3.66), being higher among boys (mean: 4.00 l; 95%CI 3.97- 4.04) than girls (mean: 3.30; 95%CI 3.27- 3.32). Lung function was directly associated with socioeconomic status at birth and at 15 years of age. Particularly among boys, trajectories of socioeconomic status were also related to lung function. Boys who were always poor had, on average, 0.31 l lower FEV1 as compared to those who were never poor; FVC among them was, on average, 0.37 l lower than the values for those who were never poor. Equivalent values for girls were 0.18 l and 0.17 l, respectively (Table 1).

**Table 1:** Descriptive values (mean and SD) for lung function (in liters) according to the socioeconomic status

Variable	Boys (n = 1969)		Girls (n = 2036)	
	
	FEV1	FVC	FEV1	FVC
Socioeconomic status at birth (tertiles)	P < 0.001	P < 0.001	P < 0.001	P < 0.001
Lowest	3.35 (0.66)	3.87 (0.75)	2.88 (0.44)	3.25 (0.53)
Intermediate	3.47 (0.68)	4.02 (0.79)	2.92 (0.42)	3.29 (0.49)
Highest	3.61 (0.62)	4.19 (0.71)	3.02 (0.44)	3.38 (0.51)
Socioeconomic status at 15 y (tertiles)	P < 0.001	P < 0.001	P < 0.001	P < 0.001
Lowest	3.33 (0.67)	3.85 (0.77)	2.84 (0.45)	3.21 (0.52)
Intermediate	3.45 (0.64)	3.99 (0.73)	2.94 (0.42)	3.30 (0.51)
Highest	3.62 (0.66)	4.19 (0.75)	3.02 (0.42)	3.38 (0.49)
Socioeconomic trajectory from birth to 15 y	P < 0.001	P < 0.001	P < 0.001	P < 0.001
Always poor	3.27 (0.65)	3.79 (0.76)	2.82 (0.45)	3.19 (0.55)
Non-poor, poor	3.41 (0.71)	3.94 (0.80)	2.87 (0.43)	3.24 (0.47)
Poor, non-poor	3.45 (0.66)	3.96 (0.76)	2.94 (0.41)	3.30 (0.51)
Never poor	3.58 (0.64)	4.16 (0.74)	3.00 (0.43)	3.36 (0.50)

**Total**	**3.46 (0.66)**	**4.00 (0.76)**	**2.93 (0.44)**	**3.30 (0.52)**

1993 Pelotas (Brazil) Birth Cohort, 2008.

FEV1: Forced expiratory volume; FVC: forced vital capacity

The results of the unadjusted and adjusted associations between lung function and socioeconomic status are presented in Tables 2 (boys) and 3 (girls). In the unadjusted analyses, all the associations were highly significant. When adjusted for the main variables (age, height, weight and BMI for each gender), the association between lung function (FEV1 and FVC) and socioeconomic status at birth and at 15 years of age remained significant, although with less strength. Socioeconomic trajectories from birth to 15 years of age were no longer associated with lung function among boys, but remained significant among girls.

**Table 2:** Unadjusted and adjusted association between lung function (mL) and socioeconomic status among boys (N = 1969)

Variable	Unadjusted		Model 1*		Model 2**	
	
	FEV1 (mL)	FVC (mL)	FEV1 (mL)	FCV (mL)	FEV1 (mL)	FVC (mL)
	
	Beta (SE)	Beta (SE)	Beta (SE)	Beta (SE)	Beta (SE)	Beta (SE)
Socioeconomic status at birth (tertiles)	P < 0.001†	P < 0.001†	P = 0.03†	P = 0.03†	P = 0.74†	P = 0.40†
Lowest	0	0	0	0	0	0
Intermediate	117 (36)	151 (41)	16 (27)	26 (30)	-15 (31)	32 (34)
Highest	266 (36)	323 (42)	61 (28)	68 (31)	12 (32)	28 (35)
Socioeconomic status at 15 y (tertiles)	P < 0.001†	P < 0.001†	P = 0.01†	P = 0.03†	P = 0.60†	P = 0.84†
Lowest	0	0	0	0	0	0
Intermediate	127 (37)	139 (42)	44 (26)	34 (28)	2 (38)	42 (42)
Highest	294 (36)	344 (41)	64 (26)	64 (29)	-17 (37)	14 (42)
Socioeconomic trajectory from birth to 15 y	P < 0.001	P < 0.001	P = 0.10	P = 0.11	P = 0.99	P = 0.74
Always poor	0	0	0	0	0	0
Non-poor, poor	142 (55)	143 (63)	12 (38)	-14 (42)	3 (60)	14 (66)
Poor, non-poor	177 (45)	169 (52)	54 (32)	19 (35)	0 (46)	6 (51)
Never poor	313 (39)	365 (45)	63 (28)	59 (31)	-9 (42)	42 (46)

1993 Pelotas (Brazil) Birth Cohort, 2008.

FEV1: Forced expiratory volume; FVC: forced vital capacity

* Adjusted for age, height, weight and BMI at 15y separated for each gender.

** Adjusted for Model 1 plus parental variables (smoking and wheezing when adolescents were 11 years of age) and adolescent variables (wheezing, smoking, pubertal stage, physical activity, skin color, allergy) separated for each gender.

† P-value of linear trend.

Among boys, when further adjustments were made (Model 2), all associations with socioeconomic-related variables lost statistical significance. Among girls, socioeconomic status at birth and socioeconomic trajectories lost statistical significance, whereas socioeconomic level at 14-15 years of age remained statistically significant (Tables 2 and 3).

**Table 3:** Unadjusted and adjusted association between lung function and socioeconomic status among girls (N = 2036)

Variable	Unadjusted		Model 1*		Model 2**	
	
	FEV1 (mL)	FVC (mL)	FEV1 (mL)	FCV (mL)	FEV1 (mL)	FVC (mL)
	
	Beta (SE)	Beta (SE)	Beta (SE)	Beta (SE)	Beta (SE)	Beta (SE)
Socioeconomic status at birth (tertiles)	P < 0.001†	P < 0.001†	P = 0.002†	P = 0.06†	P = 0.08†	P = 0.25†
Lowest	0	0	0	0	0	0
Intermediate	41 (23)	38 (28)	30 (20)	26 (23)	15 (20)	14 (23)
Highest	144 (24)	138 (28)	63 (21)	44 (24)	36 (21)	27 (24)
Socioeconomic status at 15 y (tertiles)	P < 0.001†	P < 0.001†	P < 0.001†	P = 0.002†	P = 0.001†	P = 0.005†
Lowest	0	0	0	0	0	0
Intermediate	104 (23)	92 (27)	56 (19)	34 (22)	60 (24)	37 (28)
Highest	183 (24)	174 (28)	92 (20)	72 (23)	87 (25)	83 (30)
Socioeconomic trajectory from birth to 15 y	P < 0.001	P < 0.001	P < 0.001	P = 0.06	P = 0.004	P = 0.07
Always poor	0	0	0	0	0	0
Non-poor, poor	49 (35)	43 (41)	36 (29)	27 (33)	48 (38)	33 (45)
Poor, non-poor	117 (30)	108 (35)	73 (25)	54 (28)	71 (30)	45 (36)
Never poor	174 (25)	164 (30)	87 (21)	63 (24)	96 (27)	83 (32)

1993 Pelotas (Brazil) Birth Cohort, 2008.

FEV1: Forced expiratory volume; FVC: forced vital capacity

* Adjusted for age, height, weight and BMI at 15y.

** Adjusted for Model 1 plus parental variables (smoking and wheezing when adolescents were 11 years of age) and adolescent variables (wheezing, smoking, pubertal stage, physical activity, skin color, allergy).

† P-value of linear trend.

Table 4 shows the mediation analysis using FEV1 as the outcome variable for boys. By far, height was the main explanatory variable of the association between socioeconomic status and lung function. For example, among boys, a change of 79.2% in the regression coefficient was observed comparing a model with socioeconomic status alone and a model that also incorporate height. The R^2 ^of these two models were, respectively, 2.7 and 49.2. Inclusion of an extra 10 variables did not result in additional relevant changes. The R^2^, for example, increase only from 49.2 to 53.6 if we compare a model with SES and height only with another which also includes an additional 10 variables.

**Table 4:** Mediation analysis of the association between socioeconomic status at birth and of FEV1 (mL) at 15 years of age among boys (N = 1969)

Variable (model)	Beta (SE)	Change in beta value (%)	p-value	R²
Socioeconomic status at birth (1)	132.0 (17.9)	0	< 0.001	2.7
1 + height (2)	27.5 (13.2)	-79.2	0.037	49.2
2 + weight (3)	20.1 (13.0)	-84.8	0.124	50.6
3 + skin color (4)	9.0 (12.0)	-93.2	0.485	52.2
4 + pubertal stage (5)	7.8 (15.6)	-94.1	0.617	51.5
5 + allergy (6)	10.7(15.8)	-91.9	0.498	50.9
6 + parental smoking in 2004 (7)	7.0 (17.9)	-94.7	0.696	52.9
7 + parental wheezing in 2004 (8)	6.6 (18.0)	-95.0	0.715	53.1
8 + adolescent smoking (9)	6.5 (18.0)	-95.1	0.717	53.1
9 + adolescent wheezing (10)	8.3 (17.9)	-93.7	0.645	53.6
10 + age (11)	7.6 (17.9)	-94.2	0.665	53.7
11 + physical activity (12)	7.7 (17.9)	-94.2	0.667	53.6

1993 Pelotas (Brazil) Birth Cohort, 2008.

In table 5 the equivalent analysis is presented for girls. The pattern was not the same observed among boys. First, the inclusion of height in the model changed the coefficient by 55.7%, as compared to 79.2% among boys. Second, the explained variance (R^2^) of the models was consistently lower than those observed among boys. Third, the association between SES and lung function remained statistically significant even after inclusion of all mediating variables in the model. In spite of these differences, the R^2 ^in the model with height only (29.5%) was just slightly different from the one obtained by the full model (34.9%).

**Table 5:** Mediation analysis of the association between socioeconomic status at birth and of FEV1 (mL) at 15 years of age among girls (N = 2036)

Variable (model)	Beta (SE)	Change in beta value (%)	p-value	R²
Socioeconomic status at birth (1)	70.0 (11.8)	0	< 0.001	1.7
1 + height (2)	31.0 (10.1)	-55.7	0.002	29.5
2 + weight (3)	30.6 (9.8)	-56.3	0.002	33.0
3 + skin color (4)	16.8 (9.8)	-76.0	0.087	35.0
4 + pubertal stage (5)	18.5 (10.0)	-73.6	0.064	35.1
5 + allergy (6)	19.1 (10.0)	-72.7	0.057	35.2
6 + parental smoking in 2004 (7)	28.9 (11.8)	-58.7	0.014	35.2
7 + parental wheezing in 2004 (8)	29.3 (11.9)	-58.1	0.014	34.6
8 + adolescent wheezing (9)	30.1 (11.9)	-57.0	0.012	34.9
9 + adolescent smoking (10)	31.3 (12.0)	-55.3	0.009	34.9
10 + age (11)	31.3 (12.0)	-55.3	0.009	34.9
11 + physical activity (12)	31.3 (12.0)	-55.3	0.009	34.9

1993 Pelotas (Brazil) Birth Cohort, 2008.

## Discussion

The association between SES and lung function is still inconclusive, in spite of several studies from various countries. It is possible that lung function reflects the effects of early life exposures that influence lung growth and development. A reduced supply of nutrients to the fetus may result in low birth weight and, depending on the timing, may result in specific detrimental effects to growing organs such as the lungs. Maternal smoking during pregnancy and maternal nutrition throughout life course may be also affect lung development. Postnatal factors such as acute respiratory infections, morbidities (e.g. asthma) and smoking during adolescence are possible determinants of lung function. Although all these factors are related to socioeconomic status, the most plausible explanation for an association between low socioeconomic status and poor lung function is multifactorial.

SES at birth, in adolescence and its trajectory from birth to adolescence were inversely associated with lung function in both adolescent boys and girls in our paper. After adjustment for mediating variables, coefficients were largely reduced, particularly among boys, and the main predictor of change in coefficients was the inclusion of height in the models. Therefore, an important fraction of the SES differentials in lung function is explained by height, which is in accordance with one of the postulated factors associated with lower SES that affect lung function: poor nutrition [9].

Lawlor and colleagues reported that poverty was associated with a reduction of 160 mL in FEV1 and 110 mL in FVC among older adults aged 60-79 years of age in the United Kingdom [10]. In the United States, Jackson and coworkers found that the rate of decline in lung function in 5 years was higher among the poor [11]. In addition, the authors found a negative effect of childhood SES on adult lung function [11]. Also in the United States, a study showed that low education was the only predictor of more rapid FEV1 decline among nonsmokers [1]. Taken together, these studies clearly suggest SES differentials in lung function throughout the lifespan.

Our study adds to the current knowledge in several aspects. First, we show that SES trajectories do matter in terms of later lung function. As compared to subjects who changed their SES position over time, those who were 'never poor' or 'always poor' consistently presented the highest and lowest lung function results, respectively. Second, we present the first low and middle-income prospective data on this association. Third, we explore the reasons why low income individuals present impaired lung function and most of the association with SES is explained by lower height. That is, lower income individuals are shorter (mean height: 161.2 ± 7.9 cm) than those with higher income (mean height: 164.6 ± 8.3 cm), and likewise smaller lungs that are in general terms proportional to the body size.

Among boys, the anthropometric effect was so strong that incorporation of height and weight in the model completely removed the SES differentials in lung function. Among girls, although a similar pattern was observed and thus a marked reduction in the coefficients was seen, the association with SES remained statistically significant even in the model with 11 different predictors, suggesting a more complex impact of SES on lung function involving body size, composition, and other factors.

Some methodological aspects of our study need to be discussed. A possible limitation is that SES groups were defined on the basis of family income, and a certain degree of misclassification is expected because of the difficulties at collecting income data. Another limitation is that data on smoking, a well known determinant of lung function, was collected by means of self-report, and we have previously shown that in early adolescence, self-reported data on smoking presents poor agreement with cotinine measurements [12]. The use of SES data in two periods is a positive aspect of our analyses, because it allowed us to investigate SES trajectory instead of focusing only on a static point in time. The high follow up rate and the high quality of the spirometric tests also need to be highlighted.

## Conclusions

In summary, low income adolescents from Brazil present impaired lung function as compared to the better off, and this is largely explained by height. Similar studies in other cohorts are needed to confirm our findings. Nutritional interventions targeting low income individuals may have a long-term positive impact on lung function [13].

## List of abbreviations

BMI: body mass index; CI: confidence interval; FEV1: Forced expiratory volume in one second in liters; FVC: Forced vital capacity also in liters; R²: coefficient of determination; SD: standard deviation; SES: Socioeconomic status

## Competing interests

The authors declare that they have no competing interests.

## Authors' contributions

AMBM, SCD, RPP and PCH participated in all phases of the study (conception, design, analyses of data and writing of manuscript); RBN and CLPA contributed with the acquisition of the data or the analysis and interpretation of such information; FCW and JMM provided substantial involvement in its revision prior to submission. Dr. AMB Menezes is the guarantor of the paper. All authors contributed to early drafts of this manuscript and approved its final version.

## Pre-publication history

The pre-publication history for this paper can be accessed here:

http://www.biomedcentral.com/1471-2458/11/596/prepub
